# Sema4D silencing increases the sensitivity of nivolumab to B16-F10 resistant melanoma via inhibiting the PI3K/AKT signaling pathway

**DOI:** 10.7717/peerj.15172

**Published:** 2023-04-19

**Authors:** Zhuo Zhang, Duoli Zhang, Fang Wang, Jiao Liu, Yuhong Sun, Songyot Anuchapreeda, Singkome Tima, Zhangang Xiao, Suwit Duangmano

**Affiliations:** 1 Department of Medical Technology, Faculty of Associated Medical Sciences, Chiang Mai University, Chiang Mai, Thailand; 2 Department of Pharmacology, School of Pharmacy, Southwest Medical University, Luzhou, China; 3 Department of Pharmacy, Affiliated Hospital of Southwest Medical University, Luzhou, China

**Keywords:** Sema4D, Melanoma, Nivolumab, PI3K/AKT, PD-1 inhibitor resistance

## Abstract

Melanoma is a common skin tumor that causes a high rate of mortality, especially in Europe, North America and Oceania. Immunosuppressants such as anti-PD-1 have been used in the treatment of malignant melanoma, however, nearly 60% of patients do not respond to these treatments. Sema4D, also called CD100, is expressed in T cells and tumor tissues. Sema4D and its receptor, Plexin-B1, play crucial roles in the process of immune regulation, angiogenesis, and tumor progression. The role of Sema4D in melanoma with anti-PD-1 resistance is poorly understood. Through a combination of molecular biology techniques and *in silico* analysis, the role of Sema4D in improving anti-PD-L1 sensitivity in melanoma was explored. The results showed that the expression of Sema4D, Plexin-B1 and PD-L1 was significantly increased in B16-F10R cells. Sema4D knockdown synergizes with anti-PD-1 treatment, cell viability, cell invasion and migration were significantly decreased, while the apoptosis was increased, the growth of tumors on the mice was also inhibited. Mechanistically, bioinformatics analysis revealed that Sema4D is involved in the PI3K/AKT signaling pathway; the downregulation of p-PI3K/PI3K and p-AKT/AKT expression were observed in Sema4D knockdown, therefore, nivolumab resistance is related to Sema4D and Sema4D silencing can improve sensitivity to nivolumab via inhibition of the PI3K/AKT signaling pathway.

## Introduction

Melanoma, which is prevalent in Europe and North America, has been on the rise in recent years (Rodriguez-Cerdeira et al., 2017). Melanoma is the most aggressive and fatal skin cancer, which can easily metastasize to the whole body through lymph and blood vessels. If the tumor metastasizes to distant and visceral organs, the 5-year survival rate is only 15–20% (Eggermont, Spatz & Robert, 2014; Huang & Zappasodi, 2022). Surgery is an important treatment for melanoma, chemotherapy such as paclitaxel and cisplatin are also effective, and immunotherapy such as anti-PD-1/PD-L1 also plays an important role in changing the survival rate of melanoma (Miller & Mihm Jr, 2006). Because cutaneous melanoma is often associated with UV exposure and elevated tumor mutational load, it is one of the most immunogenic of all cancer types and one of the most effective for immunotherapy (Melixetian, Pelicci & Lanfrancone, 2022).

Immune checkpoints such as programmed cell death protein-1(PD-1) and programmed death-ligand 1(PD-L1) play an important role in immunotherapy. PD-L1 is distributed in tumor cell and PD-1 is generally distributed in T-cells, when PD-L1 bind to its receptor PD-1, triggers inhibitory signaling to attenuate T cell activity (Sun, Mezzadra & Schumacher, 2018). PD-1 or PD-L1 inhibitors such as nivolumab and pembrolizumab can prevent the binding of PD-1 to PD-L1, thus restoring T cell activity and killing melanoma, non-small lung cancer (NSCLC) and other tumors (Larkin et al., 2015; Ribas et al., 2015; Weber et al., 2015). However, clinical evidence indicated that there are only 30–40% of patients receiving PD-1 or PD-L1 inhibitors could achieve a complete response (Hugo et al., 2016). Furthermore, there are a growing subset of responders acquired resistance within two years after treatment in melanoma (Robert et al., 2015a; Robert et al., 2015b). Therefore, it is an urgent to find new biomarkers with predictive value for melanoma or other solid tumors that are ineffective or resistance in the treatment of PD-1 or PD-L1 inhibitors, and ultimately improve the efficacy of PD-1 or PD-L1 inhibitors.

Semaphorins is involved in regulating tumor growth and metastasis. Especially in the tumor microenvironment, the disturbance between different cell types can control the development and progression of cancer (Lontos et al., 2018; Valentini et al., 2021). Semaphorin 4D (Sema4D) is a member of the semaphorins, which plays a important role in the progress of immunoregulatory, platelet inactivation, angiogenesis stimulation and bone formation regulation (Lontos et al., 2018). Sema4D is upregulated in most tumor tissues, such as prostate, colon, breast, melanoma, head, and neck carcinomas (Liu et al., 2015). As a ligand of Plexin-B1, Sema4D plays important roles in tumor cell proliferation, survival, and migration (Ch’ng & Kumanogoh, 2010).

The phosphatidylinositol 3-kinase/protein kinase B (PI3K/AKT) pathway is involved in the occurrence and development of melanoma. lncRNA OR3A4, GRB2 and Hey1 can promote melanoma migration and invasion *via* this signaling pathway (Shayan et al., 2017). When PI3K combines with GRB2, it will be phosphorylated and recruited to the cell membrane, then AKT phosphorylatedand acts on its target genes to regulate a variety of cellular processes (Yu et al., 2006).

In this study, we use a combination of molecular biology techniques and bioinformatics to explore the roles of Sema4D in anti-PD-1 resistance melanoma. We demonstrated for the first time that Sema4D is involved in anti-PD-1 resistance in melanoma. Moreover, Sema4D deficiency can overcome resistance to anti-PD-1 therapy and the mechanism is related to PI3K/AKT signaling pathway.

## Materials and Methods

### Cell culture and B16-F10 resistance cell line establishment

293T cells, B16-F10 were purchased by Nanjing Cobioer Biotechnology Co., LTD (Nanjing, China), cultured at 37 °C in 5% CO_2_ in RPMI-1640 (Life Technologies, Invitrogen, Canada) with 10% FBS (Gibco, Billings, MT, USA). The combination of high dose shock and gradually increasing dose was applied to establish the cell lines insensitive to nivolumab (anti-PD-1). The B16-F10 cell line in the logarithmic growth phase were cultured with nivolumab (AbMole Bioscience Inc., Shanghai, China) 10 ng/mL and TIL at a 1:5 ratio. After 24 h, the solution was changed, and dead cells were discarded. The remaining cells were then cultured with RPMI-1640 without nivolumab, when the remaining cells have stabilized, then cultured with nivolumab 25 ng/ml and repeat these steps until the cells could grow continuously and stably in the medium with nivolumab 50 ng/mL, and then cultured drug-free for 1 month, B16-F10 resistance cell line (B16-F10R) were obtained. B16-F10R also needs with nivolumab 5 ng/mL to maintain the resistance (Maeda, Lafreniere & Jerry, 1991).

### Tumor infiltrating lymphocyte (TIL)

The tumors were removed from the Balb/c mice, about 2–3 cubic millimeters in size, and cultured with two mL complete medium in 24 well plates. The complete medium included 6,000 IU/mL IL-2 which purchased from Novartis (Basel, Switzerland), RPMI-1640 and 2.05 mM L-glutamine were the major components and purchased from Thermo Fisher Scientific (Waltham, MA, USA), there is also a need to add 10% heat-inactivated human AB serum (Omega Scientific, Westlake Village, CA, USA), 55 µM 2-mercaptoethanol and 10 mM of HEPES Buffer (Mediatech, Hsinchu, Taiwan), gentamicin (50 µg/mL), penicillin (100 I.U./mL), streptomycin (100 µg/mL) were purchased from Invitogen and prevent infection (Arneth, 2019).

### Western blotting

We investigated the expression of PD-L1, Sema4D, Plexin-B1, PI3K, p-PI3K, AKT and p-AKT. Antibodies against these proteins were purchased from Abcam Shanghai Trading Co., LTD and cell signaling technology, Inc. (Shanghai, China). Total protein was extracted and BCA protein detection kit (Beyotime, Jiangsu, China) was used to evaluate the protein concentration. after electrophoresis, transfer membrane, antibody incubation process, the dilute concentration of primary antibody is PD-L1(1:500), Sema4D(1:1000), Plexin-B1(1:1000), PI3K(1:100), p-PI3K(1:1000), AKT(1:1000), p-AKT(1:500), an ECL chromogenic substrate was applied for detecting the signals (Beyotime, Jiangsu, China).

### RT-qPCR

Total RNA was obtained by processing the samples with TRIzol reagent and Rnase-free DNase Set (Beyotime, Jiangsu, China). The synthesis of complementary DNA was performed using a cDNA synthesis kit (Thermo Fisher Scientific, Waltham, MA, USA). Finally, RT-qPCR analysis was performed using an ABI PRISM 7900-HT sequence detection system (Applied Biosystems, Waltham, MA, USA). For RT-qPCR, the following primers were used.

PD-L1:5′-AGAACCTGGCTGCACCTAAC-3′(F),

5′-GAGAAGGTCAAACCGCCTCA-3′(R);

Plexin-B1:5′-AAGCCCAGCCTACTAACAACC-3′(F),

5′-CAGCCCCACTGTACTGACTG-3′(R);

Sema4D:5′-TCAAAGCAGACGGAATGCCTA-3′(F),

5′-CCCCAACCATGACTGATGTGTA-3′(R);

GAPDH:5′-TTGTCATGGGAGTGAACGAGA-3′(F),

5′-CAGGCAGTTGGTGGTACAGG-3′(R).

### shRNA and expression plasmid transfection

Short hairpin RNA (shRNA) targeting Sema4D and the lentivirus containing this shRNA were designed and synthesized by Genepharm Co. (Shanghai, China), sh-sema4D (5′-GGATGGGACTG TCTATGATGT-3′) and the control NC-sema4D (5′-TTCTCCGAACGTGTCACGT-3′). The shuttle plasmid and three other packaging plasmids (pGag/Pol, pRev, pVSV- G) were co-transfected into 293T cells to generate a lentivirus expressing Sema4D shRNA or a control lentivirus expressing shRNA. Cells were inoculated into 6-well plates at 1 × 10^5^ and infected with lentivirus while 5 µg/mL of Polybrene was added. The culture media was changed after 24 h of infection, and the infected cells were screened with 1 µg/mL of puromycin for 7 days after the culture media change to obtain stable lentiviral shRNA expressing cell lines for subsequent experimental analysis (Moore et al., 2010).

### Sema4D overexpression

B16-F10 cells were treated with RPMI 1640 containing 10% fetal bovine serum culture medium was placed in 5% CO_2_ incubator at 37 °C, and logarithmic growth was taken long-term cells were used in the experiment. Construct Sema4D lentivirus overexpression vector LV-Sema4D and negative control LV-NC, these vectors were commissioned by Gemma Pharmaceutical Technology Co., LTD. (Shanghai, China) to synthesize. B16-F10 cell was inoculated in 24-well plates and cultured for 24 h multiplicity of infection, complete culture medium was replaced for further culture 48 h, the expression of Sema4D was detected by RT-qPCR.

### MTT

Cell viability was measured by Methyl Thiazolyl Tetrazolium (MTT) assay. MTT was dissolved in DMSO and 0.5 mg/mL MTT diluted in 1 × PBS for 4 h, Turn on Benchmark Plus microplate reader (Bio-Rad, Hercules, CA, USA) and detect absorbance at 570 nm (Algazi et al., 2020).

### Cell apoptosis analysis

Cell apoptosis were detected by AnnexinV-AlexaFluor647/PI Kit Apoptosis Assay Kit (Biotech, Minneapolis, MN, USA). 5 × 10^5^ B16-F10R cells were seeded in 10-cm culture dishes (Corning, Corning, NY, USA) and incubated overnight. 5 × 10^5^ treated cells were collected and resuspended with 500 µL binding buffer. 5 µL Annexin V/AlexaFluor647 were incubate for 5 min at room temperature, then add 10 µL propidium iodide (PI), fluorescent dyes should be protected from light. All operations are performed as per manufacturer instructions.

### Wound healing and invasion analysis

B16-F10R cells invasion and migration was analyzed by Matrigel-coated invasion chambers and scratch assays respectively. The initial number of cells cultured was 5 × 10^5^, a sterile 10 µL pipette tip was used to mark a line in the monolayer of each well, the wounds were observed at 0, 24 and 48 h under a microscope (Nikon, Japan). ImageJ software was used to measure the wound areas. Cell free migration area =(cell free area 0 h - cell free area 24h)/cell free area 0 h (Ji et al., 2018). 5 × 10^5^ cells were placed into the Matrigel-coated invasion chambers (Corning, USA) to incubation, the upper and lower cultures were separated by polycarbonate membranes, and the cells under study were planted in the chamber, after 48 h, the top of Matrigel and invading cells were fixed and stained with 0.1% crystal violet. The microscope (Nikon, Japan) was used to photograph invading cells, and 3 fields were randomly selected and counted each time.

### Animal and tumorigenesis

Balb/c mice with 12-week-old were purchased from Chendu dossy experimental animals CO., LTD (SCXK (Chuan) 2020-030, Chengdu, China). The mice were fed unrestricted diet and water intake, and were kept under specific pathogen-free conditions in a laboratory where a 12-hour light/dark cycle was strictly enforced (SYXK(Chuan) 2018-065, Luzhou, China). For each group, at least 5 mice were used (Sanmamed et al., 2015). Tumor volumes were measured on 1, 3, 6, 9, 12, 15 and 18 days with caliper, at days 18, mice were sacrificed, tumor weight was measured, tumor volumes were calculated with the formula: tumor volume = 0.52 × length × width^2^ (mm^3^) .

### Data collection

Melanoma gene expression profiling data were extracted from the Cancer Genome Consortium (TCGA) database using the TCGAbiolinks software package. Expression profiles were converted to transcripts per million mapped reads per kilobase exon model (TPM) and normalized by log2.

### Weighted co-expression network analysis

To explore the signaling pathways involved in Sema4D, gene co-expression network analysis (WGCNA) was first conducted to determine genes with significant change between high- and low-expression of Sema4D through the WGCNA package on R project. Topological overlapping measurement was utilized to identify modules most associated with Sema4D expressions. Correlation between modules and traits is calculated by Spearman correlation analysis.

### Protein-protein interaction

To explore the potential functions played by Sema4D in various biological processes, we constructed Sema4D-related protein-protein interaction network by STRING (https://cn.string-db.org/).

### Gene set variation analysis

Gene set variation analysis was conducted to reveal the molecular mechanisms under the different Sema4D expression populations in melanoma. The h.all.v7.5.1.symbols.gmt gene set was used to enrichment analysis and was downloaded from MSigDB (https://www.gsea-msigdb.org/gsea/index.jsp).

### Functional enrichment analysis of Sema4D

To explore the mechanism of action of Sema4D involved in PD-1 inhibitor resistance, enrichment analysis on hub genes was performed. “ClusterProfiler” package in R project was utilized to identify the signaling pathways involved in Sema4D within TCGA cohort (which contains 471 melanoma samples).

### Statistical analysis

GraphPad Prism V.7.00 software was utilized for statistical analysis. Different statistical methods were selected according to different experimental purposes, compare the differences between two groups, we chose Student’s t test, one-way ANOVA was used to compare the differences between the overall differences. *P* < 0.05 is considered to be statistically significant.

## Results

### Sema4D, Plexin-B1 and PD-L1 upregulation in nivolumab resistance B16-F10 cells

**Figure 1:**
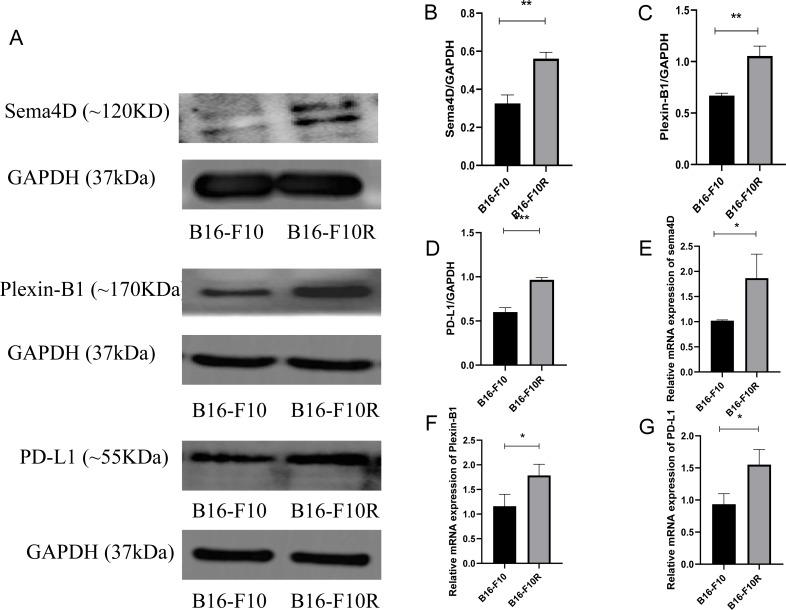
Sema4D, Plexin-B1 and PD-L1 expression in B16-F10 and B16-F10R cells. Cells were derived into B16-F10R group (B16-F10R cells) and B16-F10 (B16-F10 cells), 24 h after cell culture, Sema4D, Plexin-B1 and PD-L1 expression were detected. (A) Protein bands of Sema4D, Plexin-B1 and PD-L1. (B) Ratio to GAPDH of Sema4D. Image J V1.8.0 software was used to measure the gray value of the value of the protein band, and then the relative density value was obtained by comparing the value of GAPDH with the target protein, *p* = 0.0026. (C) Ratio to GAPDH of Plexin-B1, *p* value = 0.0018. (D) Ratio to GAPDH of PD-L1, *p* value = 0.0016. (E) mRNA expression of Sema4D, *p* value = 0.037. (F) mRNA expression of Plexin-B1 , *p* = 0.0312. (G) mRNA expression of PD-L1, *p* = 0.0212. Compared with B16-F10 group, Sema4D and Plexin-B1 were significantly upregulated in B16-F10R group. ^∗^ is *p* < 0.05, ^∗∗^ is *p* < 0.01, ^∗∗∗^ is *p* < 0.001. Repeat the experiment three times (*n* = 3), t test was used for statistical analysis.

To investigate the mRNA and protein expression of Sema4D, Plexin-B1and PD-L1 in sensitive cell line and insensitive cell line of melanoma, mRNA and protein expression of Sema4D, Plexin-B1 and PD-L1 of B16-10 and B16-F10R were detected. Compared with B16-10 group, mRNA and protein expression of Sema4D, Plexin-B1and PD-L1 were significantly upregulation in B16-F10R group (Figs. 1A–1G).

### Sema4D downregulation inhibit PD-L1 expression upon anti-PD-1 therapy

**Figure 2:**
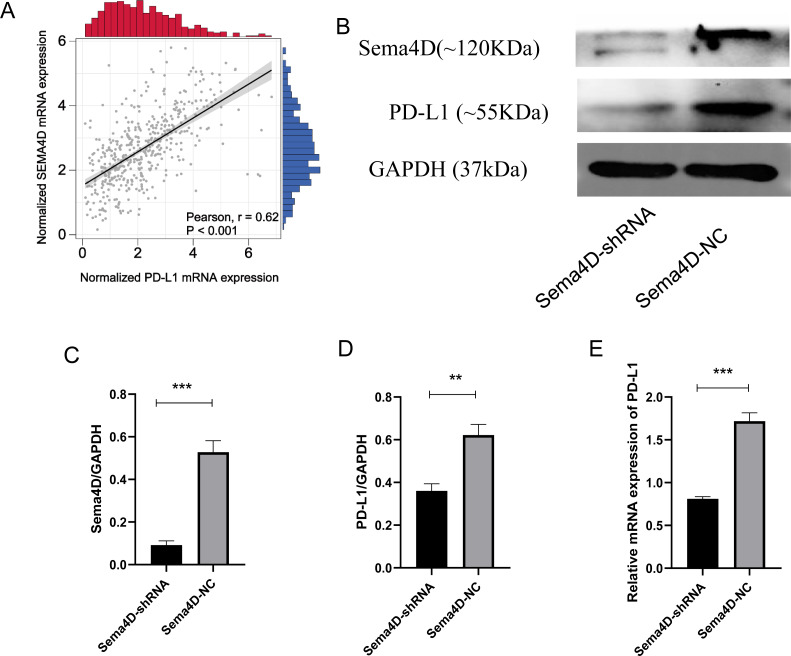
Sema4D silencing decrease PD-L1 expression. B16-F10R Cell were derived into Sema4D-shRNA group and Sema4D-NC group, co-cultured with TIL at a 1:5 ratio and nivolumab 50 ng/ml 24 h, collect the cell to detect the expression of PD-L1. (A) Correlation between PD-L1 and Sema4D (B) Protein bands of PD-L1and Sema4D. (C) Ratio to GAPDH of Sema4D, *p* = 0.0002. (D) Ratio to GAPDH of PD-L1, *p* = 0.0016. (E) mRNA expression of PD-L1. Compared with Sema4D-NC group, ^∗∗^ is *p* < 0.01, ^∗∗∗^ is *p* < 0.001. The experiment was repeated three times, *n* = 3, t test was used for statistical analysis.

We analyzed the correlation of PD-L1 and Sema4D with bioinformatics, it showed that PD-L1 and Sema4D are positively correlated (Fig. 2A) in TCGA-cohort. Compared with Sema4D-NC group, protein expression of Sema4D in Sema4D-shRNA group was significantly downregulated, it indicated that Sema4D silencing is success (Figs. 2B and 2C). Compared with Sema4D-NC group, mRNA and protein expression of PD-L1 in Sema4D-shRNA group was significantly downregulated after nivolumab treatment, which was consistent with the results of bioinformatics (Figs. 2B, 2D and 2E). Therefore, Sema4D silencing significantly repressed the mRNA and protein expression of PD-L1.

### Sema4D repression renders B16-F10R cells sensitive to PD-1 treatment

**Figure 3:**
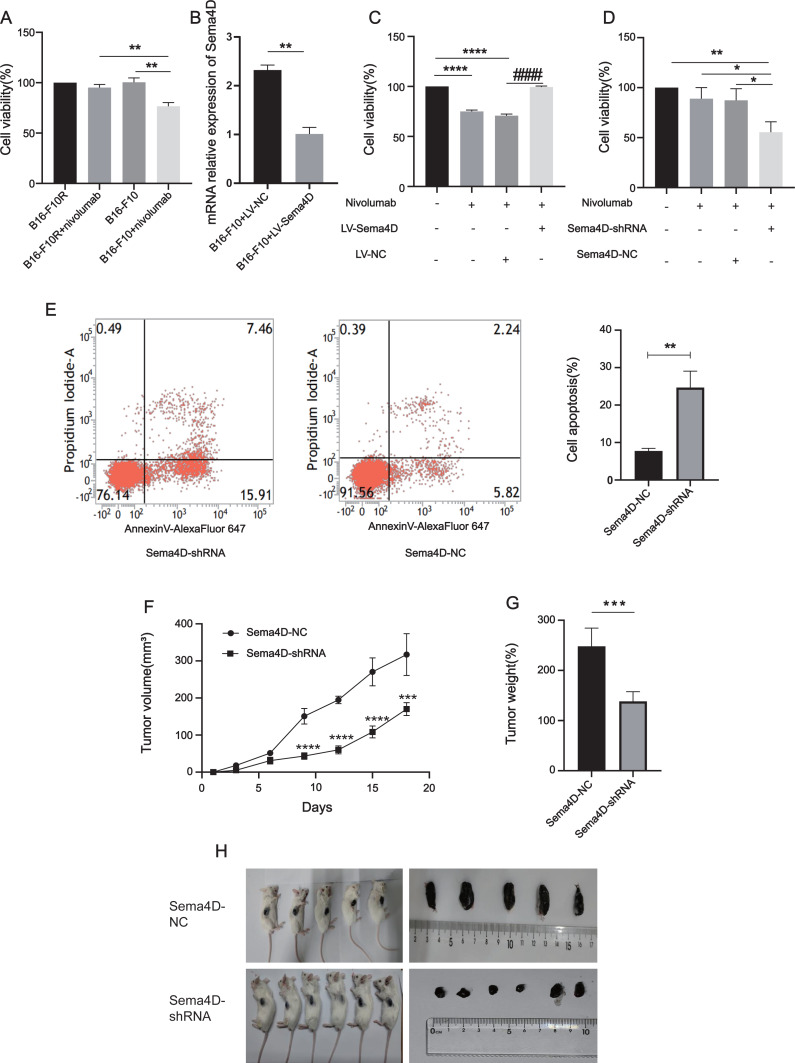
Sema4D repression renders B16-F10R cells sensitive to nivolumab treatment. (A) Effects of nivolumab on cell viability of B16-F10 and B16-F10R. B16-F10 cells were derived into B16-F10 and B16-F10+nivolumab group, B16-F10R cells were derived into B16-F10R and B16-F10R+nivolumab groups, B16-F10 and B16-F10R groups were as control group without treatment, B16-F10+nivolumab and B16-F10R+nivolumab groups were treatment with 50 ng/mL nivolumab and co-cultured with TIL at a 1:5 ratio (Van den Berg et al., 2020). Compared with the B16-F10+nivolumab group, ^∗∗^ is *p* < 0.01, *n* = 3, one-way ANOVA was used to compare the differences. (B) RNA expression of Sema4D overexpression. Cells were derived into B16-F10+LV-Sema4D and B16-F10+LV-NC group, compared with the LV-NC group, ** is *p* < 0.01, *n* = 3, *t*-test was used to compare the differences. (C) Effects of nivolumab on B16-F10 cell viability after Sema4D overexpression. B16-F10 cells were derived into B16-F10, B16-F10+nivolumab, B16-F10+LV-Sema4D and B16-F10+LV-NC group, B16-F10 is control group, another 3 groups were treatment with 50ng/mL nivolumab and co-cultured with TIL at a 1:5 ratio, B16-F10+LV-Sema4D group was also treated with overexpression Sema4D, B16-F10+LV-NC group was also treated with Sema4D negative control. Compared with B16-F10 group, **** is *p* < 0.0001; Compared with B16-F10+LV-NC group, #### is *p* < 0.0001, *n* = 3, one-way ANOVA was used to compare the differences. (D) Effects of nivolumab on B16-F10R cell after Sema4D silencing. B16-F10R cells were derived into B16-F10R, B16-F10R+nivolumab, Sema4D-shRNA and Sema4D-NC group , B16-F10R is control group, another 3 groups were treatment with 50ng/mL nivolumab and co-cultured with TIL at a 1:5 ratio, Sema4D-shRNA group was knockdown Sema4D, and Sema4D-NC group was Sema4D-shRNA negative control group. Compared with Sema4D-shRNA group, * is *p* < 0.05, ** is *p* < 0.01, *n* = 3, one-way ANOVA was used to compare the differences. (E) B16-F10R cell apoptosis rate after treatment with nivolumab. B16-F10R cells were derived into Sema4D-shRNA group and Sema4D-NC group, the treatment is the same as C. Cell apoptosis was detected byPI and Annexin V/Alexa Fluor 647 double staining, Alexa Fluor 647 was a fluorescent dye binding with Annexin V, reflecting early apoptosis of cells. Compared with Sema4D-NC group, ** is *p* < 0.01, *n* = 3, *t*-test was used to compare the differences. (F) Tumor volume of knockdown Sema4D with nivolumab treatment. Balb/C mice were derived into Sema4D-shRNA group (*n* = 6) and Sema4D-NC group(*n* = 5), B16-F10R-Sema4D-shRNA cells and B16-F10R-Sema4D-NC cells were intradermal injected into the right side of mice, then intraperitoneally injected nivolumab 10 mg/kg once at day 1, *t*-test was used to compare the differences. (G) Tumor weight of knockdown Sema4D with nivolumab, *t*-test was used to compare the differences. Compared with Sema4D-NC group, *** is *p* < 0.001, **** is *p* < 0.0001. (H) Mouse melanoma specimen. Major marks represent cm on the scale.

To explore the relationship between Sema4D downregulation and nivolumab treatment efficacy in B16-F10 and B16-F10R cells, we investigated the cell viability and cell apoptosis. First, we observe the effects of nivolumab on the cell viability of B16-F10 and B16-F10 resistant cell lines. Compared with the B16-F10 cell lines, nivolumab had inhibitory effect on B16-F10 cell lines (*p* = 0.0018), it is suggested that nivolumab has inhibitory effect on sensitive cell lines. Compared with the B16-F10+nivolumab group, the inhibitory effect of nivolumab on drug-resistant cell lines was decreased significantly (*p* = 0.0025), it is suggested that nivolumab has weak inhibitory effect on resistance cell lines (Fig. 3A). Next, we explored the effects of nivolumab on the cell viability of Sema4D overexpressed in B16-F10 cells. First, the mRNA expression of Sema4D was detected, and the results showed that Sema4D expression of LV-Sema4D group was increased significantly compared with the LV-NC group(*p* = 0.0083), it indicates that Sema4D transfection is successful (Fig. 3B). Through MTT, compared with LV-NC group, nivolumab have little inhibitory effect on B16-F10 cells with Sema4D overexpression (*p* < 0.0001), this suggest that Sema4D overexpression could weaken nivolumab effect, so Sema4D was related to nivolumab sensitivity to B16–F10 cells. (Fig. 3C). We further explored the effect of nivolumab on the cell viability of Sema4D knockdown B16-F10R cell lines, the results showed Sema4D downregulation potentiates anti-PD-1 treatment efficacy in B16-F10R cells. Compared with Sema4D-NC group, the cell viability of Sema4D-shRNA group in combination with nivolumab was decreased significantly (*p* = 0.0186) (Fig. 3D) and rate of apoptosis cell in Sema4D-shRNA group after treatment with anti-PD-1 was notably increased (*p* = 0.0027) (Fig. 3E). Finally, we tested the effect of nivolumab on Sema4D-knockdown melanoma in mice. *In vivo* experiment, we transplanted B16-F10R cells in the mice and observed the size and weight of tumors treated with nivolumab, tumor volume and weight were significantly delayed in Sema4D-shRNA group mice as compared to Sema4D-NC group mice (Figs. 3F, 3G and 3H).

### Sema4D repression potentiates the effect of anti-PD-1 on cell invasion and migration

**Figure 4:**
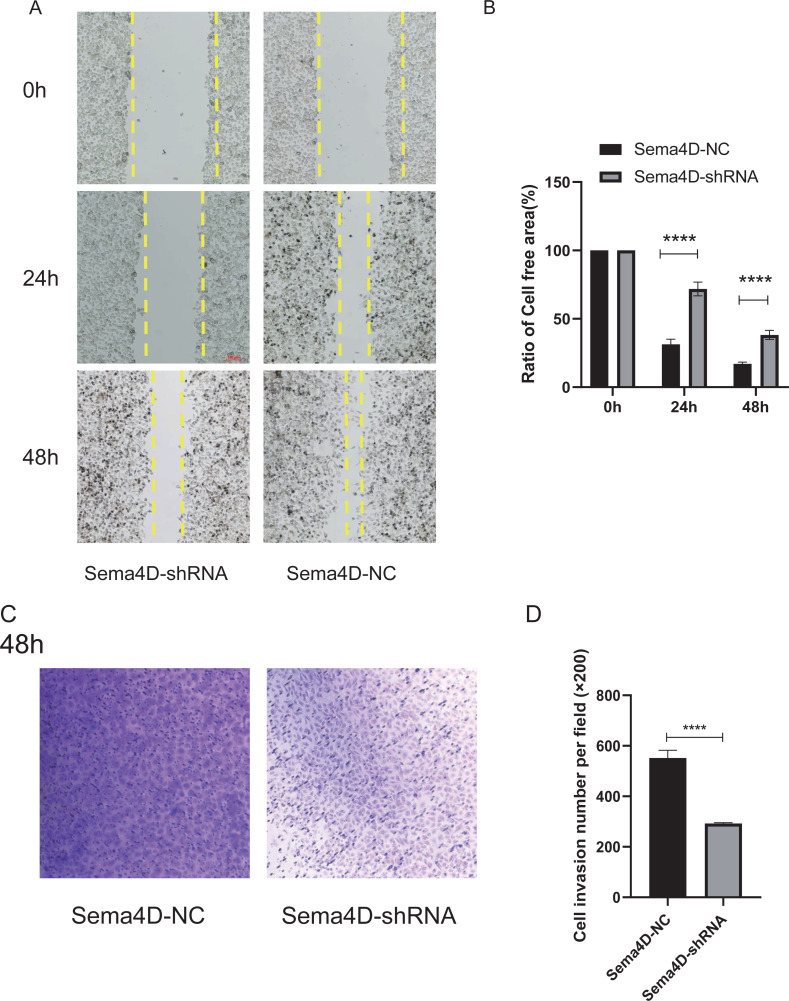
Sema4D deficiency potentiates nivolumab inhibitory effects on B16-F10R cell invasion and migration. B16-F10R cells were derived into Sema4D-shRNA group and Sema4D-NC group, co-cultured with TIL at a 1:5 ratio and nivolumab 50 ng/ml, cell invasion and migration were detected. (A) Cell migration. Representative image of cell migration in the absence and presence of shRNA Sema4D at 0 h, 24 h, and 48 h. The scratched areas were measured in three random fields in each group. Wound healing analysis showed a significant difference in the cell-free area of Sema4D-shRNA group was significantly wider than that of Sema4D-NC group at 24 and 48 h (*P* < 0.0001). (B) Ratio of cell free area. (C) Chamber invasion of cells. Chamber invasion analysis showed a significant difference lower number of invasive cells in Sema4D-shRNA group than Sema4D-NC group (× 100) at 48 h. (D) cell invasion number per field. Compared with Sema4D-NC group,^∗∗∗∗^ is *p* < 0.0001. The expression was repeated three times, *n* = 3.

The effect of Sema4D repression with anti-PD-1 treatment on B16-F10R cell invasion and migration was analyzed by Matrigel-coated invasion chambers and scratch assay. The results showed that when knockdown of Sema4D, cell migration of Sema4D-shRNA group was decreased (*p* < 0.0001), the cell-free area between migrating cells from Sema4D-shRNA group was wider than the gap between cells of the Sema4D-NC group (Figs. 4A and 4B). In addition, the number of invaded cells was significantly less in Sema4D-shRNA group in contrast to Sema4D-NC group (Figs. 4C and 4D), these data suggesting that lowering Sema4D could enhance the inhibitory effect of anti-PD-1 on cell migration and invasion.

### Downregulation of Sema4D inhibit PI3K/AKT expression

**Figure 5:**
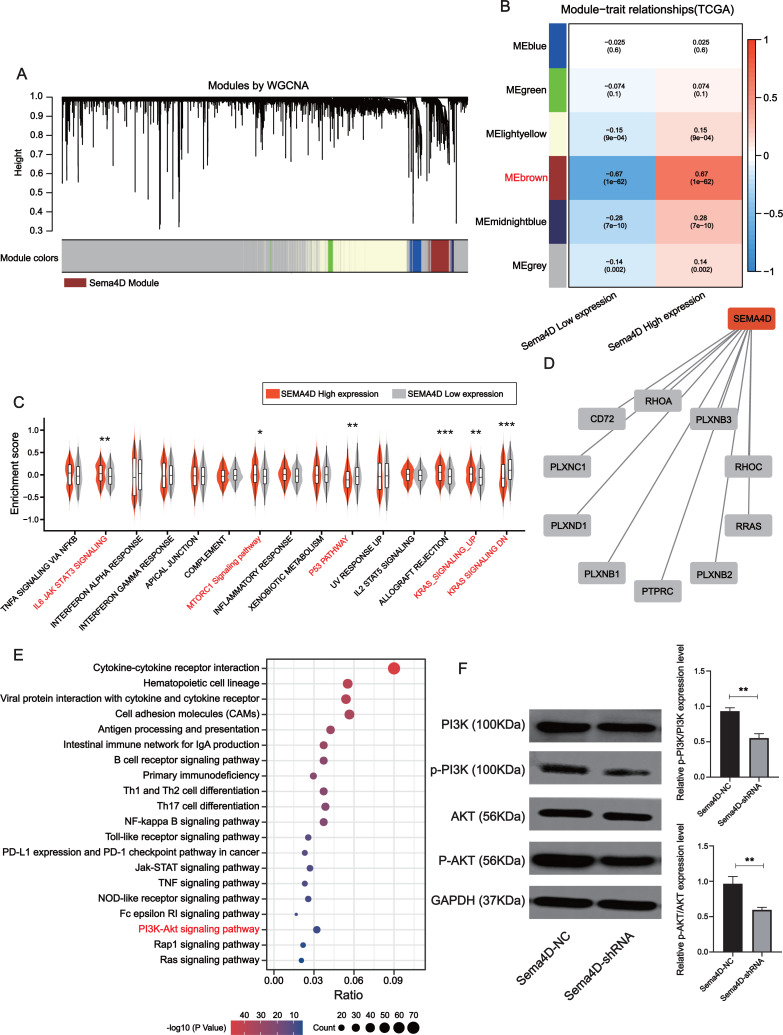
Sema4D knockdown is significantly associated with inhibition of PI3K-AKT signaling pathway. (A) Association of clustered modules with Sema4D expression. The brown module was identified as the most relevant module for Sema4D expression. (B) Module-trait associations. (C) Gene set variation analysis revealed several significantly dysregulation malignancies events between different Sema4D expression population. (D) Protein-protein interactions of Sema4D. (E) Pathway enrichment analysis showed that Sema4D is involved in the PI3K-AKT signaling pathway. (F) PI3K-AKT signaling pathway proteins expression after Sema4D knockdown. B16-F10R cells were derived into Sema4D-shRNA group and Sema4D-NC group, co-cultured with TIL at a 1:5 ratio and nivolumab 50 ng/ml, PI3K, AKT protein expression were detected. The experiment was repeated three times, *n* = 3. Western blotting showed that Sema4D was significantly associated with inhibition of PI3K-AKT signaling pathway . Compared with Sema4D-NC group, ^∗∗^ is *p* < 0.01.

The brown module was determined to be most significantly associated with Sema4D expression (*r* =  +  − 0.67, *p*-value = 1e−62), which contained a total of 778 genes (Figs. 5A–5B). Gene set variation analysis (GSVA) revealed several significantly upregulated malignancy-associated events (IL-6, JAK, STAT3, MTORC1, and KRAS signaling pathways) in the Sema4D high expression group as well as downregulation of p53 signaling (Fig. 5C). Sema4D was significantly associated with oncogenes RHOA, RRAS and immunomodulatory-related genes PTPRC and PLXNC1 (Fig. 5D). Pathway enrichment analysis revealed that Sema4D is involved in multiple immune-related pathways as well as the PI3K/AKT pathway (Fig. 5E). Numerous studies have demonstrated that excessive activation of PI3K/AKT signaling pathway is associated with excessive proliferation of tumor cells, inhibition of apoptosis and attenuation of the efficacy of immune checkpoint inhibitors (Zhang, Richmond & Yan, 2022). The expression of PI3K/AKT protein was explored by Western blot. p-PI3K/PI3K, p-AKT/AKT of Sema4D-shRNA group were decreased than those of Sema4D-NC group, which suggesting aberrant activation of the PI3K/AKT signaling pathway in the Sema4D-NC group (Fig. 5F).

## Discussion

Immunotherapy is one important method for melanoma treatment, when alone or in combination with anti-PD-1 and anti-PD-L1 may improve survival rates (Simeone, Grimaldi & Ascierto, 2015). Nivolumab is a PD-1 inhibitor with high affinity to PD-1 (Hirano et al., 2005). However, the ineffectiveness or resistance of anti-PD-1 to melanoma is a serious problem. In general, anti-PD-1 resistance are mainly derived into acquired resistance and innate resistance (Bridgeman et al., 2014; Marincola et al., 2000; Bronte et al., 2005; O’Donnell et al., 2017). The causes of anti-PD-1 or anti-PD-L1 resistance to melanoma are very complex and not fully understood. This study intends to elucidate that Sema4D may be involved in the sensitivity of melanoma to nivolumab therapy *via* PI3K/AKT signaling pathway.

Nivolumab is a PD-1 inhibitor, PD-1 exists in T cells while PD-L1 exists in tumor cells, so the effect of nivolumab on tumor cells requires the presence of T cells. B16-F10 is a melanoma cell line derived from C57BL/6J mice, but tumor formation can also be inoculated subcutaneously in Balb/c mice (Colombo et al., 2019). In this experiment, Tumor Infiltrating Lymphocyte (TIL) was obtained from Balb/c mice and added to B16-F10 or B16-F10R cell to simulate tumor microenvironment. B16-F10 resistance cells were implanted subcutaneously in Balb/c mice by subcutaneous injection to observe tumor volume and weight.

Sema4D expressed on many cancer cells, Plexin-B1 also expression in cancer such as ovarian, melanoma, when Sema4D combined with Plexin-B1, it correlates with tumor immune infiltration, angiogenesis, and tumor progression in melanoma (Ch’ng & Kumanogoh, 2010; Lu et al., 2021). To understand the expression of Sema4D in melanoma resistance to anti-PD-1, B16-F10 resistant cell lines was established, after administered 50 ng/ml nivolumab, the killing ability of nivolumab was significantly weaker than that of B16-F10 cell lines, this means that resistant cell lines are established successfully. Then we explored mRNA and protein expression of Sema4D and Plexin-B1 in B16-F10 cell lines and B16-F10R cell lines, our results revealed that the mRNA and protein expression of Sema4D and Plexin-B1 in drug-resistant cell lines were overexpressed. Therefore, we provide the evidence for the first time that Sema4D and Plexin-B1 is related to anti-PD-1 resistance of melanoma.

To further analyzed the relationship between Sema4D and nivolumab, Sema4D were overexpression in B16-F10 cells and Sema4D expression was silencing by shRNA in B16-F10R cells. It was observed that overexpression of Sema4D can attenuates the therapeutic effect of nivolumab in B16-F10 cells and downregulation of Sema4D will increase the sensitivity of B16-F10R cell lines to nivolumab. In addition, we also investigated the effects of Sema4D knockdown on B16-F10R cells in response to nivolumab, downregulation of Sema4D also increased the inhibiting the growth of tumor cells and reducing cell invasion and migration. As reported by Rezaeepoor that silencing of Sema4D serve as a therapeutics target for the suppression of invasion, migration (Rezaeepoor et al., 2021). A study conducted by Rashidi illustrated silencing of Sema4D can elevated apoptosis rate of SW48 cells in response to 5-FU treatment (Rashidi et al., 2020). Moreover, we verified the enhancement of anti-PD-1 sensitivity by Sema4D knockdown *in vivo* xenograft models, mice treated with nivolumab after Sema4D knockdown showed significantly slower tumor growth. Taken together, these finding demonstrated that Sema4D deficiency can potentiates nivolumab treatment efficacy.

We further analyzed the correlation between Sema4D and PD-L1 expression in anti-PD-1 resistance. It has been demonstrated that the presence of PD-L1 determines the response to anti-PD-1/PD-L1 therapy (Sunshine et al., 2017), regorafenib can promotes antitumor immunity *via* inhibiting PD-L1 expression in melanoma (Wu et al., 2019). JAK1/2 mutation leads to the lack of reactive PD-L1 expression, which is involved in the resistance of PD-1/PD-L1 inhibitors (Shin et al., 2017). In this study, we evaluated PD-L1 expression in B16-F10, B16-F10R cell and B16-F10R cell knockdown with Sema4D-shRNA, the results showed PD-L1 expression in B16-F10R was increased and Sema4D knockdown group is decrease, these suggest that the effects of Sema4D knockdown on Nivolumab in B16-F10R cell lines may be related to the decrease of PD-L1 expression. However, which way contributes to the PD-L1 downregulation after knockdown Sema4D remains unclear and needs further investigation.

PI3K is phosphorylated by binding to GRB2, and AKT phosphorylates target genes to regulate cell function (Pu et al., 2021). Sema4D and plexin-B1 is associated with osteosarcoma, in which PYK2-PI3K-Akt pathway is activated to promote tumor progression (Li et al., 2021). To explore the relationship between Sema4D and PI3K/AKT signaling pathways in anti-PD-1 resistance, we detected the expression of PI3K/AKT protein, in B16-F10R cell, p-PI3K/PI3K, p-AKT/AKT expression were significantly increased, when inhibited the Sema4D, p-PI3K/PI3K, p-AKT/AKT expression of Sema4D-shRNA group were decreased, these shown that Sema4D can regulate PI3K/AKT expression and activity. Bioinformatics analysis revealed that Sema4D is involved in the PI3K/AKT signaling pathway which is activated and plays important roles such as being involved in the development and occurrence in melanoma and it also activate receptor tyrosine kinases (Davies, 2012). However, whether the relationship between Sema4D and nivolumab sensitivity is related to JAK-STAT signal pathway and other signal pathways needs further study.

In conclusion, we explored the role of Sema4D in the process of PD-1 inhibitor resistance. In anti-PD-1 resistant cell line, upregulation of Sema4D, Plexin-B1 and PD-L1 was observed. When Sema4D was silencing, it prevented the phosphorylation of PI3K and AKT, thus inhibiting the PI3K/AKT pathway activity. Therefore, these results may have therapeutic implication and may be exploited for the development of novel treatment for anti-PD-1 resistance melanoma in the future.

##  Supplemental Information

10.7717/peerj.15172/supp-1 Supplemental Information 1 R code used in this study

10.7717/peerj.15172/supp-2 Supplemental Information 2 Author Checklist

10.7717/peerj.15172/supp-3 Data S1 Raw data

10.7717/peerj.15172/supp-4 Supplemental Information 4 Original lmage of tumor shape

10.7717/peerj.15172/supp-5 Supplemental Information 5 Uncropped Western Blots

## Additional Information and Declarations

### Data Availability

The following information was supplied regarding data availability: The bioinformatics analysis code used in this study is available in the Supplemental File.
