# Supplementary figures and images for: Sema4D silencing increases the sensitivity of nivolumab to B16-F10 resistant melanoma via inhibiting the PI3K/AKT signaling pathway
Source: PeerJ. 2023 Apr 19;11:e15172. doi: 10.7717/peerj.15172 (PMC10122458; doi:10.7717/peerj.15172)

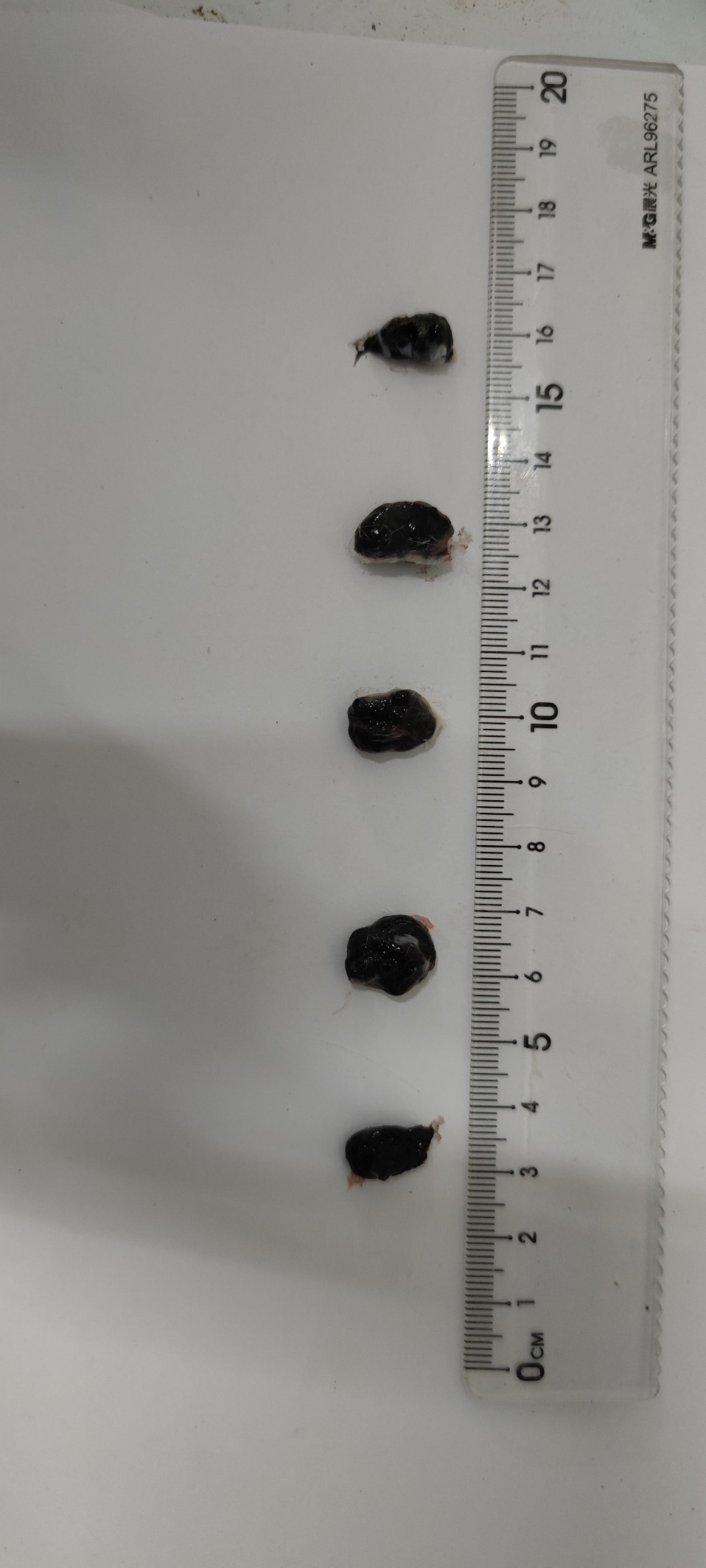

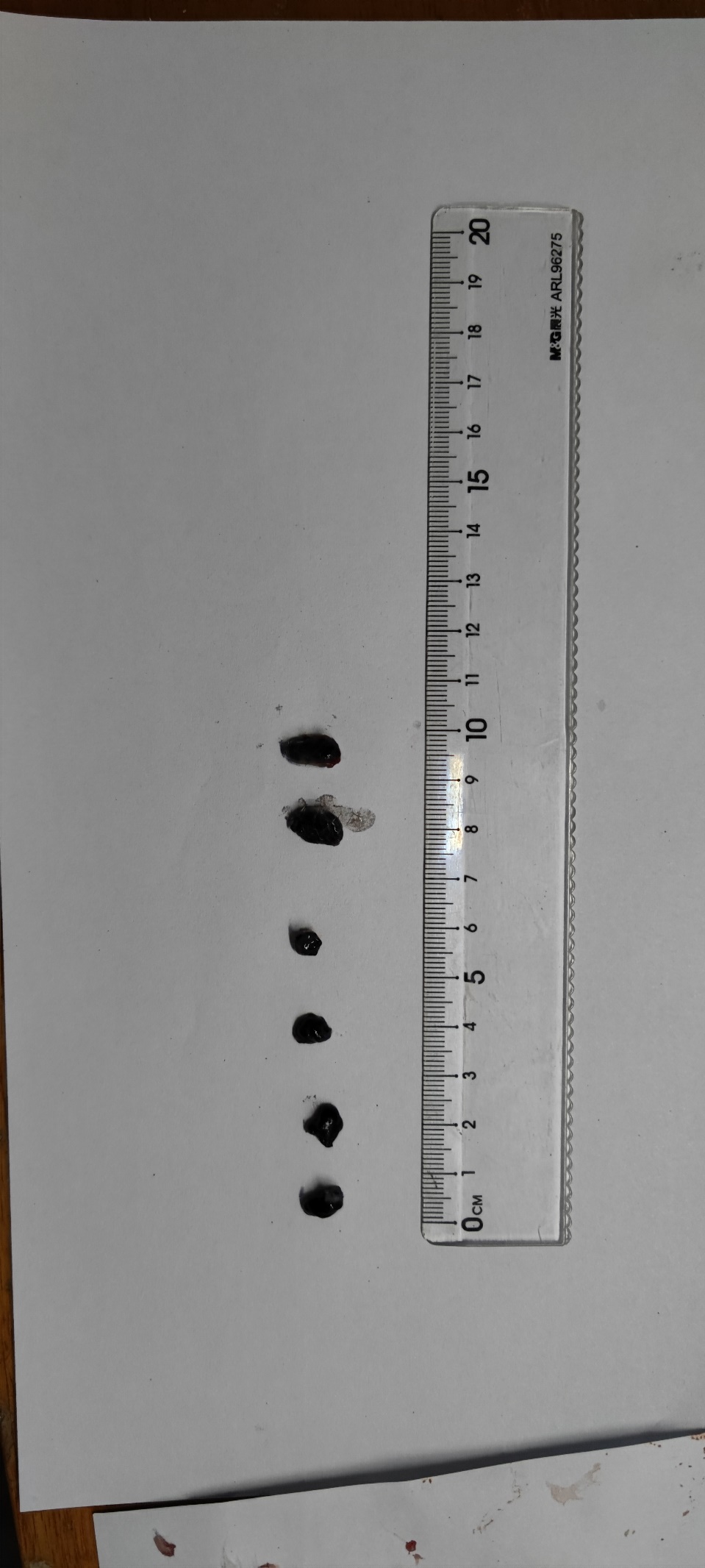

Supplement: Supplemental Information 4 [file peerj-11-15172-s004.docx]

GAPDH (37kDa)

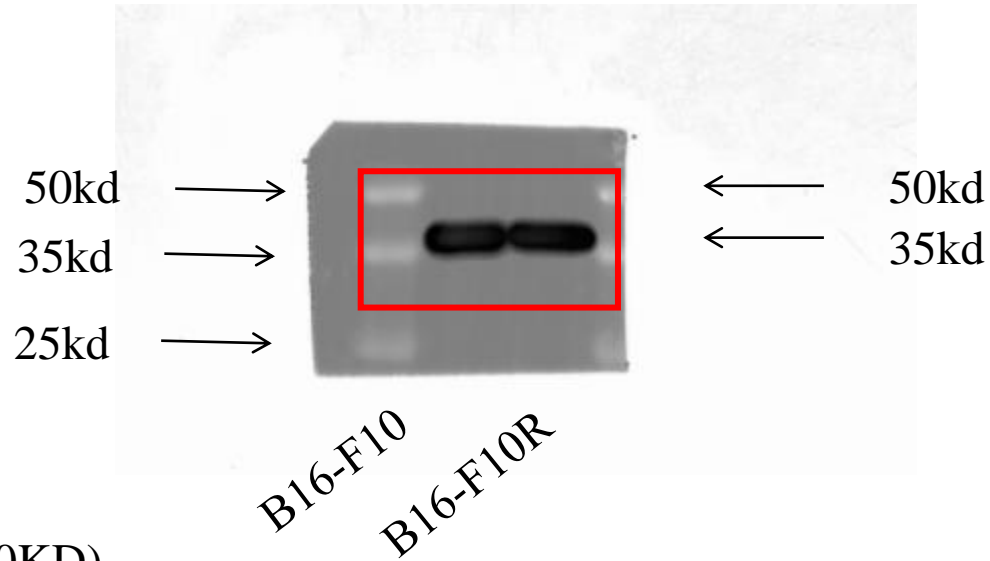

Sema4D (~120KD)

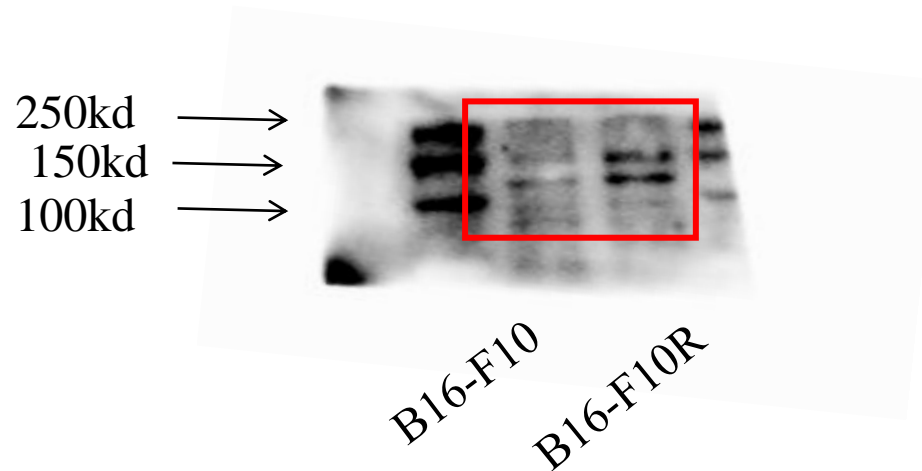

Plexin-B1 (~170KD)

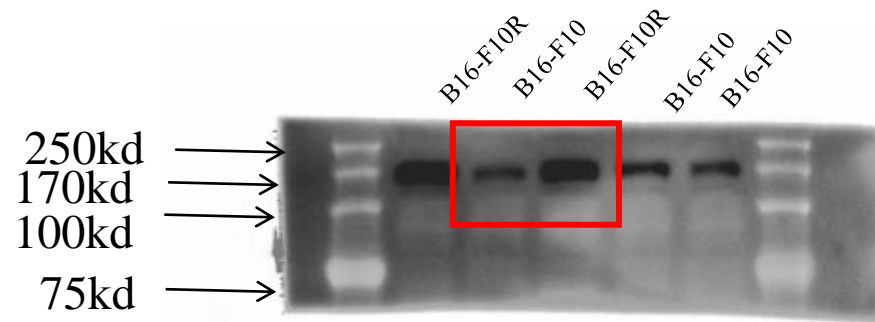

GAPDH (37kDa)

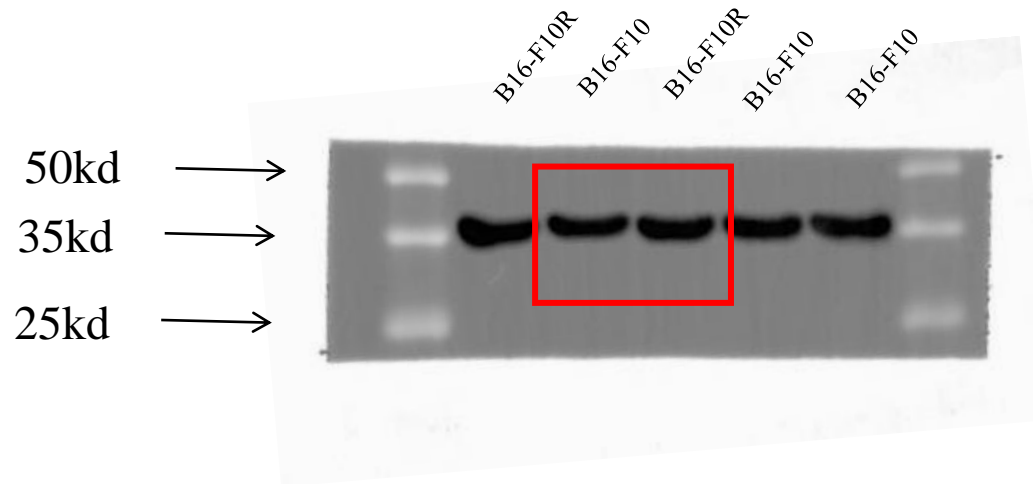

GAPDH (37kDa)

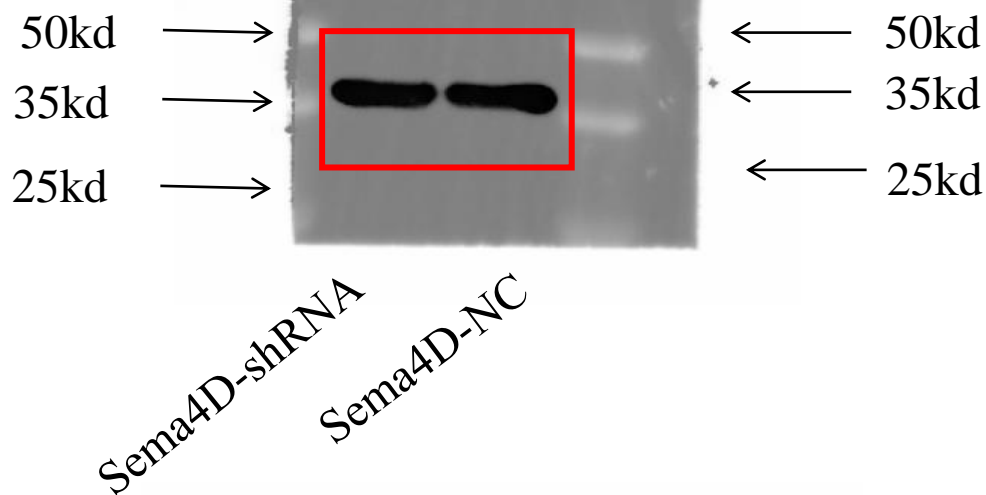

PD-L1 (~55KD)

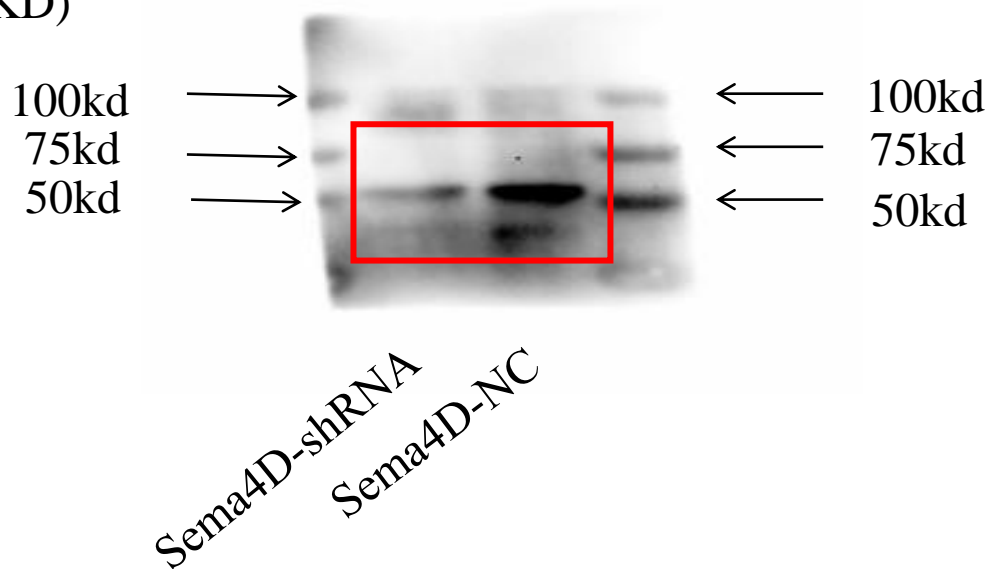

PI3K (100 kDa)

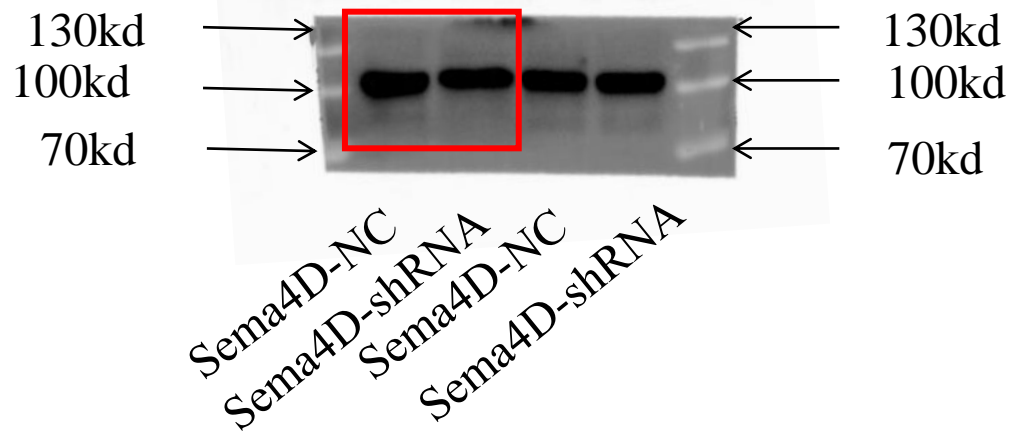

p-PI3K (100 kDa)

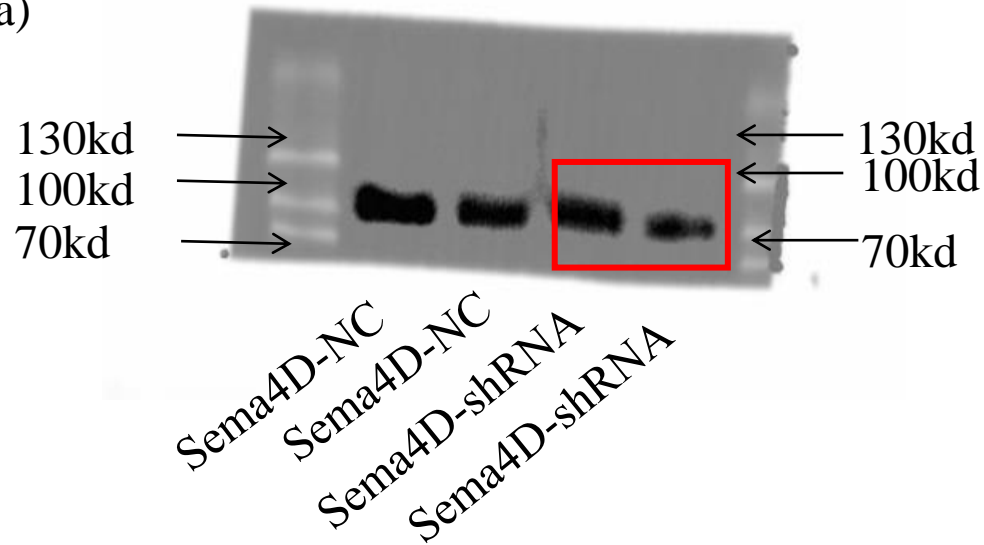

AKT (62 kDa)

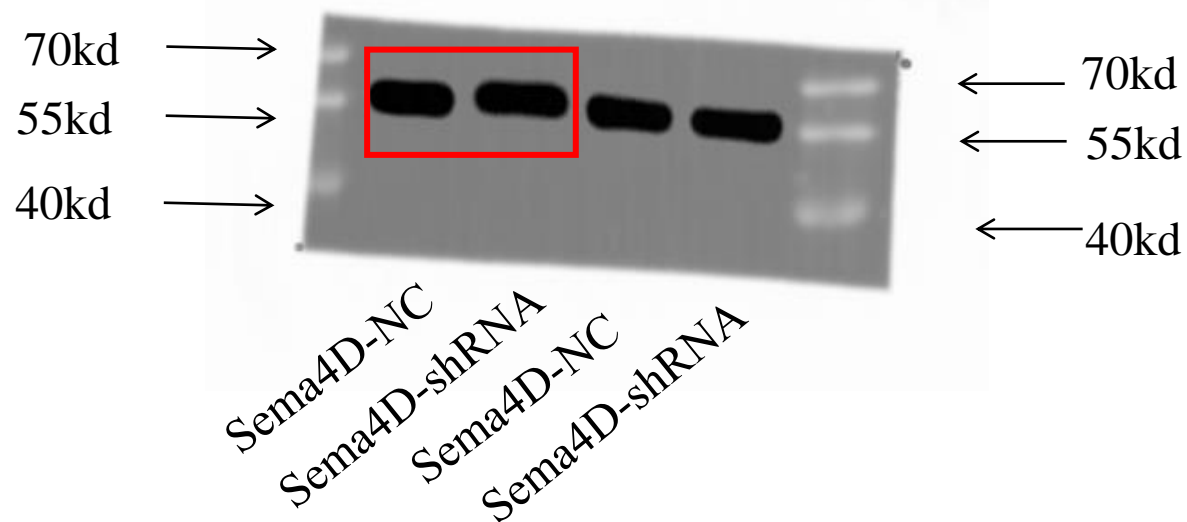

p-AKT (56 kDa)

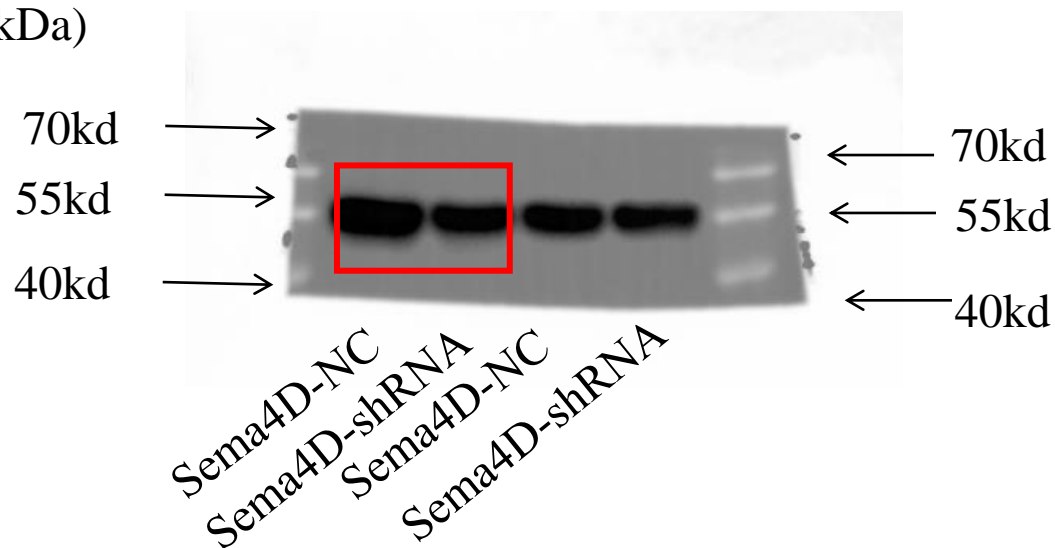

GAPDH (37kDa)

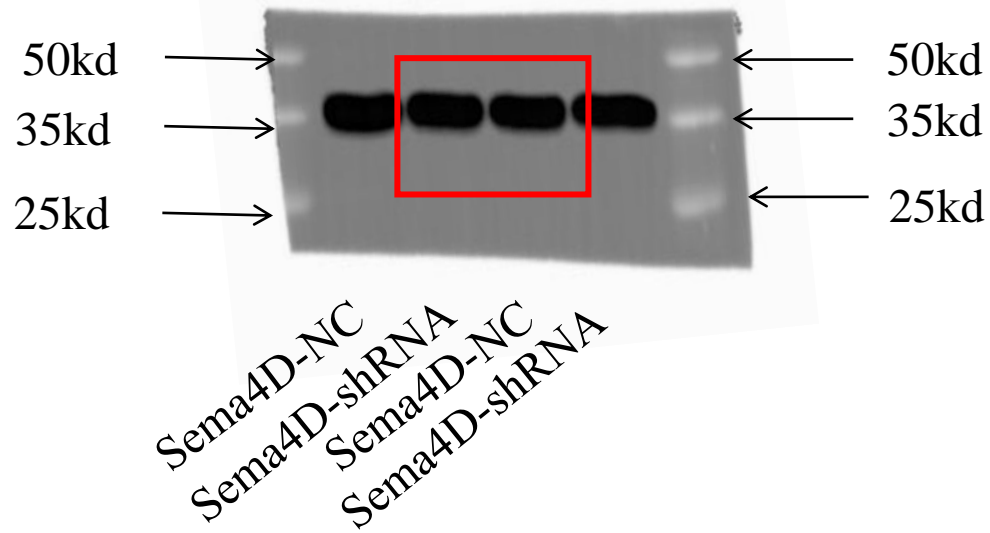

Supplement: Supplemental Information 5 [file peerj-11-15172-s005.pdf]
